# Stable Photoelectrochemical Reactions at Solid/Solid Interfaces toward Solar Energy Conversion and Storage

**DOI:** 10.1021/acs.nanolett.3c03982

**Published:** 2024-01-12

**Authors:** Kenta Watanabe, Yuhei Horisawa, Masataka Yoshimoto, Kazuhisa Tamura, Kota Suzuki, Ryoji Kanno, Masaaki Hirayama

**Affiliations:** † Department of Chemical Science and Engineering, School of Materials and Chemical Technology, Tokyo Institute of Technology, 4259 Nagatsuta-cho, Midori-ku, Yokohama 226-8501, Japan; ‡ Research Center for All-Solid-State Battery, Institute of Innovative Research, Tokyo Institute of Technology, 4259 Nagatsuta-cho, Midori-ku, Yokohama 226-8501, Japan; § Materials Sciences Research Center, Japan Atomic Energy Agency, 1-1-1 Koto, Sayo, Hyogo 679-5148, Japan

**Keywords:** Photoelectrochemistry, solid/solid interface, solid electrolyte, all-solid-state cell, solar energy conversion and storage

## Abstract

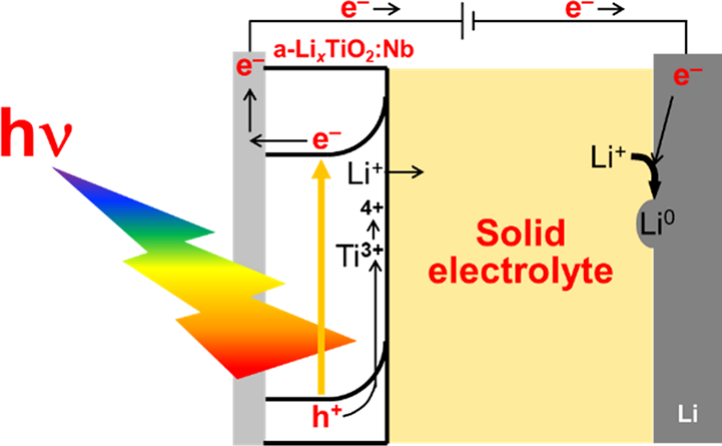

Electrochemistry has extended from reactions at solid/liquid interfaces to those at solid/solid interfaces. However, photoelectrochemistry at solid/solid interfaces has been hardly reported. In this study, we achieve a stable photoelectrochemical reaction at the semiconductor-electrode/solid-electrolyte interface in a Nb-doped anatase-TiO_2_ (a-TiO_2_:Nb)/Li_3_PO_4_ (LPO)/Li all-solid-state cell. The oxidative currents of a-TiO_2_:Nb/LPO/Li increase upon light irradiation when a-TiO_2_:Nb is located at a potential that is more positive than its flat-band potential. This is because the photoexcited electrons migrate to the current collector due to the bending of the conduction band minimum toward the negative potential. The photoelectrochemical reaction at the semiconductor/solid-electrolyte interface is driven by the same principle as those at semiconductor/liquid-electrolyte interfaces. Moreover, oxidation under light irradiation exhibits reversibility with reduction in the dark. Thus, we extend photoelectrochemistry to all-solid-state systems composed of solid/solid interfaces. This extension would enable us to investigate photoelectrochemical phenomena uncleared at solid/liquid interfaces because of low stability and durability.

Photoelectrochemistry has mainly been used in the field of photocatalysis since the discovery of the Honda–Fujishima effect.^1−9^ Electrochemistry focuses on the interactions between electrons and materials, leading to the conversion and storage of electrical energy to chemical energy. Photoelectrochemistry includes interactions between photons and electrons as well as interactions between electrons and materials. This implies that photoenergy can be stored as chemical energy via the conversion of photo-to-electric and electric-to-chemical energy in photoelectrochemistry. Therefore, the development of photoelectrochemistry is important from the viewpoint of photoenergy conversion and storage.

Recently, electrochemistry has been extended from phenomena at solid/liquid interfaces to those at solid/solid interfaces. The solid/liquid interfaces are generally paid much attention as the interaction sites between electrons and materials because standard electrochemical systems are composed of electrodes and liquid electrolytes. In particular, the electrochemistry at solid/solid interfaces has recently received considerable attention, owing to the enhancement of ionic conductors as solid electrolytes, which are used in all-solid-state batteries. The ionic conductivities of solid electrolytes, such as Li_10_GeP_2_S_12_^10^ and Li_9.54_[Si_0.6_Ge_0.4_]_1.74_P_1.44_S_11.1_Br_0.3_O_0.6_,^11^ are comparable to or higher than those of organic liquid electrolytes (∼10^–2^ S cm^–1^). Moreover, the transference numbers of carrier ions are almost 1 in solid electrolytes because the constituent elements, except the carrier ions, are fixed in the crystal lattices. This means that side reactions, such as the decomposition of electrolytes and elution of electrodes, do not continuously proceed, even if they partially and temporally occur only at electrode/electrolyte interfaces. In addition, the potential windows of solid electrolytes (>5 V) tend to be wider than those of liquid electrolytes (∼4.6 V).^12,13^ Therefore, solid electrolytes are thermodynamically more stable than liquid electrolytes. From these points of view, solid/solid interfaces may reveal phenomena that are hidden in information derived from the decomposition and elution because of unstable liquid electrolytes. Moreover, the photoelectrochemistry at solid/solid interfaces has been hardly studied, even in the fields of photocatalysis and photorechargeable batteries. Kondo and co-workers reported a p-i a-Si/SiO_*x*_/Ag_6_WO_4_/Ag_*x*_V_2_O_5_ photorechargeable battery using Ag^+^ as a carrier ion.^14^ However, it is unclear that this system photoelectrochemically works, because Ag^+^ is photocorrosively reduced to Ag^0^ regardless of electrochemical potentials.^15,16^ Thus, solid/solid interfaces are expected to be applicable to the field of photoelectrochemistry.

Thin-film type all-solid-state systems should be suitable for investigating photoelectrochemistry at solid/solid interfaces. The resistance of these systems to electronic and ionic conduction can be decreased by decreasing the thicknesses of the cathode, solid electrolyte, and anode layers. Moreover, good contacts are easily formed at the interfaces between the electrodes and electrolyte layers without voids. The decreased resistance and good contacts at the interfaces positively contribute to the progress of the electrochemical reactions. In addition, incident light easily reaches the entire electrode in the case of a thin film. We reported all-solid-state batteries with high capacities and/or retentions by fabricating thin-film type batteries.^17−22^

Photo(de)intercalation is an important topic that should be addressed as a photoelectrochemical phenomenon. However, photo(de)intercalation has never functioned steadily since the first report^23^ because of the decomposition of liquid electrolytes and elution of electrodes. Moreover, photo(de)intercalation using solid/solid interfaces has been limited to study. Therefore, the photoelectrochemical reactions at the solid/solid interfaces were expected to be constructed also from the perspective of photo(de)intercalation.

Anatase TiO_2_ (a-TiO_2_) is a suitable electrode material for photoelectrochemical reactions at solid/solid interfaces. Rutile TiO_2_ (r-TiO_2_) and a-TiO_2_ have been used as representative photocatalysts since the discovery of the Honda–Fujishima effect.^1,2,24−26^ In addition, a- and r-TiO_2_ work as hosts of Li^+^ intercalation.^27,27^ It has also been reported that the a-TiO_2_ film electrode is active for photocharging in LiClO_4_ acetonitrile solutions.^28^ Thus, a-TiO_2_ is expected to work in all-solid-state photoelectrochemical cells.

In this study, the photoelectrochemistry at solid/solid interfaces was investigated using a-TiO_2_ electrodes in thin-film all-solid-state cells. The reversibility of the redox reactions (charge–discharge) was confirmed in the dark. Then, the photoelectrochemical reactions were investigated using the all-solid-state cells.

## Fabrication of the Thin Film a-TiO_2_:Nb Electrode for the Reversible Electrochemical Reaction

The thickness of the obtained Nb-doped TiO_2_ (TiO_2_:Nb)^29−33^ prepared at 773 K was estimated to be 33 nm from the Dektak measurement. Approximately 30 nm of the thickness was estimated also from X-ray reflectivity (Figure S2). The peaks derived from a-TiO_2_ were not confirmed even in grazing incidence X-ray diffraction (GI-XRD) patterns using the synchrotron X-ray beam when the thickness of TiO_2_:Nb was about 30 nm (Figure S3). Therefore, it was difficult to identify a TiO_2_:Nb film with 30 nm thickness with XRD. The pattern derived from a-TiO_2_ was confirmed when a film with a thickness of approximately 400 nm was deposited on a SiO_2_ substrate under the same conditions, except for the deposition time (Figure S4). The peaks of the 400 nm thick a-TiO_2_:Nb showed relative intensities similar to those of the polycrystalline a-TiO_2_, suggesting that the 33 nm-thick TiO_2_:Nb films were also in the anatase polycrystalline phase.

The charge/discharge curves and capacity retention of the a-TiO_2_:Nb/LPO/Li (LPO: amorphous Li_3_PO_4_ solid electrolyte) cell were first investigated under dark conditions (Figure S5). The capacity was lower than 50 mAh g^–1^ at only the first discharge. This low capacity might have been due to the preintercalation of Li^+^ into a-TiO_2_:Nb when LPO was deposited on a-TiO_2_:Nb. After the first discharge, a-TiO_2_:Nb/LPO/Li delivered charge/discharge capacities of approximately 120 mAh g^–1^ with clear plateau regions at approximately 1.75 V. The Li^+^-(de)intercalation proceeded at 1.75 V via a two-phase reaction consisting of a phase transition between a-TiO_2_ and Li_*x*_TiO_2_ (*x* ≤ 0.55) with an anatase structure (a-Li_*x*_TiO_2_).^34−36^ This result also suggested that a-TiO_2_:Nb was successfully fabricated. Reversible charge–discharge reactions were observed during subsequent cycling, indicating highly steady Li^+^-(de)intercalation through the a-TiO_2_:Nb/LPO interface. These results indicated that the obtained a-TiO_2_:Nb film was reversibly (de)intercalated with Li^+^ at the a-TiO_2_:Nb/LPO interface under dark conditions. Thus, we concluded that the a-TiO_2_:Nb/LPO/Li cell could be applied to photoelectrochemical measurements at solid/solid interfaces from the viewpoints of stability and durability.

**Figure 1:**
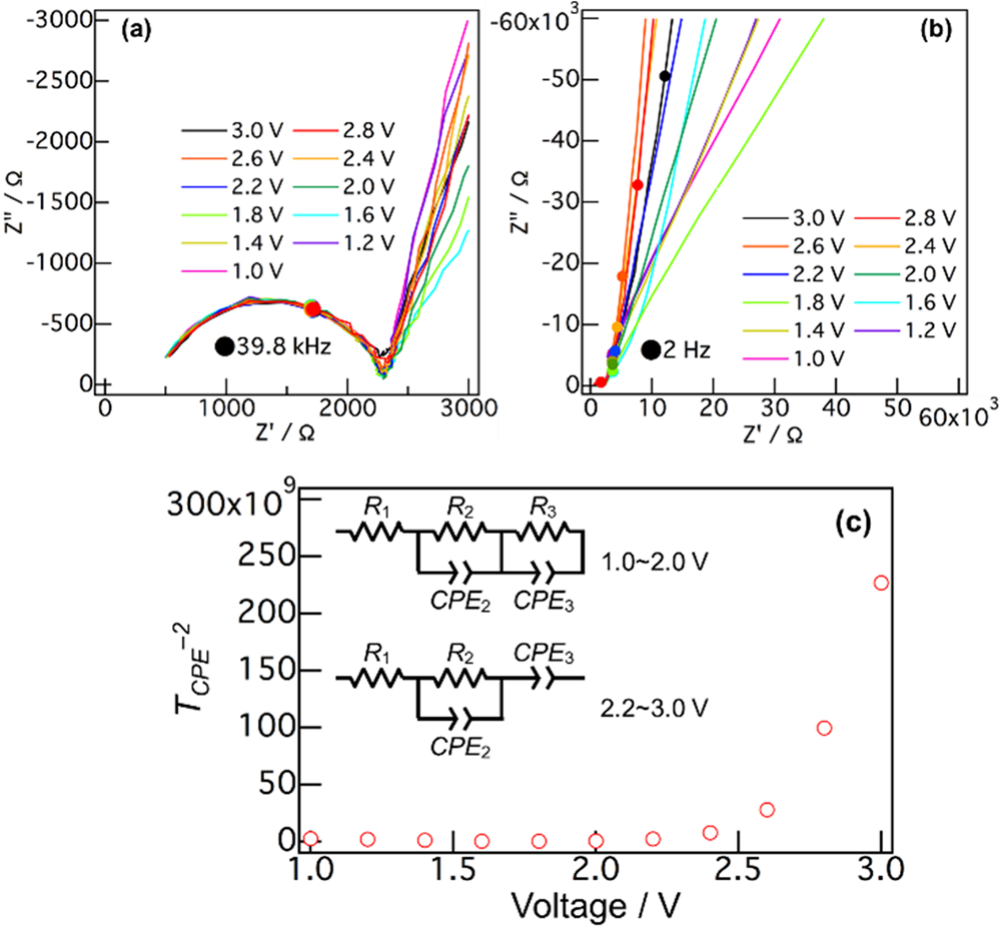
(a,b) Nyquist and (c) Mott–Schottky plots of quartz/ITO/a-TiO_2_:Nb/LPO/Li. The *T*_CPE_ values were estimated from the semicircles of the Nyquist plots. ITO means indium tin oxide current collector. [*R*_1_-*R*_2_*CPE*_2_-*CPE*_3_] and [*R*_1_-*R*_2_*CPE*_2_-*R*_3_*CPE*_3_] were applied as equivalent circuits at 2.2–3.0 and 1.0–2.0 V, respectively. *R*_1_-*R*_2_*CPE*_2_ was treated as a constant value when estimating *T*_CPE_.

It is necessary to clarify the band positions of a semiconductor electrode compared to the potential of an electrolyte because the migration of photogenerated electrons and holes is greatly affected by the conduction band minimum (CBM), valence band maximum (VBM), and Fermi level. Figure 1 shows the Nyquist and Mott–Schottky plots of a-TiO_2_:Nb/LPO/Li. The *T*_CPE_ values were estimated from the Nyquist plots. In the Mott–Schottky plot, the *T*_CPE_ (CPE constant (CPE: constant phase element)) values were shown on the *y*-axis because parts of the *T*_CPE_ values could not be converted to *C*_scl_ (capacitance of a space charge layer) values owing to the difference in the equivalent circuit. The difference between the *T*_CPE_ and *C*_scl_ affects the slopes of the fitting lines, whereas the intercepts remain relatively unchanged.^37,38^ A flat-band potential (*E*_FB_) is estimated from an intercept of a Mott–Schottky plot. Therefore, the Mott–Schottky plots obtained by using the *T*_CPE_ values could be used to estimate only the *E*_FB_. [*R*_1_-*R*_2_*CPE*_2_-*CPE*_3_] and [*R*_1_-*R*_2_*CPE*_2_-*R*_3_*CPE*_3_] (*R*: resistance) could be applied as the equivalent circuits to the Nyquist plots at 2.2∼3.0 and 1.0∼2.0 V, respectively. The semicircles in the high-frequency region (*R*_2_*CPE*_2_) did not depend on the voltage. Therefore, the LPO bulk and LPO/Li interface resistance were included in *R*_1_-*R*_2_*CPE*_2_. The semicircles in the low-frequency region were derived from the a-TiO_2_:Nb/LPO interfaces (*CPE*_3_ or *R*_3_*CPE*_3_) because they strongly depended on the voltage. Therefore, the *T*_CPE_ values were estimated from the semicircles in the low frequency region (*CPE*_3_ or *R*_3_*CPE*_3_) by fitting, on which *R*_1_-*R*_2_*CPE*_2_ was treated as the constant value. The positive slope of the Mott–Schottky plot above 2.5 V means that a-TiO_2_:Nb was an n-type semiconductor, indicating that the Fermi level was located near the CBM. The *T*_CPE_^–2^ below 2.4 V was approximately 0, indicating that the capacitance in the Helmholtz layer hardly affected the plot.^39^ Therefore, the intercept of this Mott–Schottky plot was used to estimate the *E*_FB_. The intercept of the plot was approximately 2.5 V, indicating that a-TiO_2_:Nb became the state of *E*_FB_ when a voltage of 2.5 V was applied. Thus, when a voltage larger than 2.5 V was applied, the potential of a-TiO_2_:Nb was more positive than *E*_FB_, leading to a CBM bending toward the negative potential at the a-TiO_2_:Nb/LPO interface. In contrast, the CBM bended toward a positive potential below 2.5 V.

## Investigation of the Photoelectrochemical Reaction at the a-TiO_2_:Nb/LPO Interface

**Figure 2:**
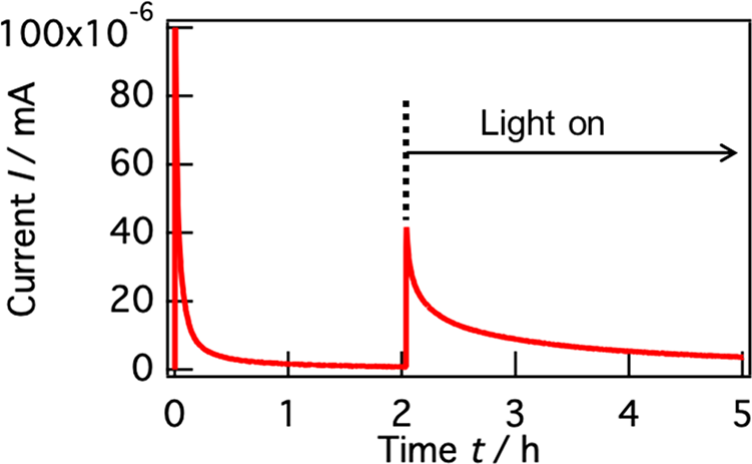
CA curve of quartz/SrRuO_3_(SRO)/a-TiO_2_:Nb/LPO/Li at 3.0 V under dark and light irradiation. The cell voltage was first raised to 3.0 V by CC charging under the dark condition. The measurement mode was switched from the CC to CA mode at 0 h.

Figure 2 shows the chronoamperometry (CA) curve of a-TiO_2_:Nb/LPO/Li at 3.0 V in the dark and under light irradiation for 2 and 3 h, respectively. The cell voltage was first raised to 3.0 V by the constant-current (CC) charge under the dark condition, and the measurement mode was subsequently switched to the CA mode. The measurement mode was switched from the CC to CA mode at 0 h, and the light was tuned on at 2 h. Under the dark condition, the current decreased with the measurement time for the first 1 h, and subsequently, an almost constant current flowed continuously for 1 h. The current drastically increased with light irradiation. The photocurrent decreased slowly over the measurement time of 3 h. All the photocurrents during 3 h were higher than the constant currents under the dark condition. Therefore, it was concluded that the photocurrent was not derived from the temporal capacitance such as the formation of electric double layers. In addition, the distance between the a-TiO_2_:Nb/LPO/Li and light source was 15 cm, which was sufficient to eliminate the thermal effect on the cell properties (Figure S6). Therefore, the photocurrent was influenced by the photoexcitation of electrons from the VB to the CB in a-TiO_2_:Nb.

**Figure 3:**
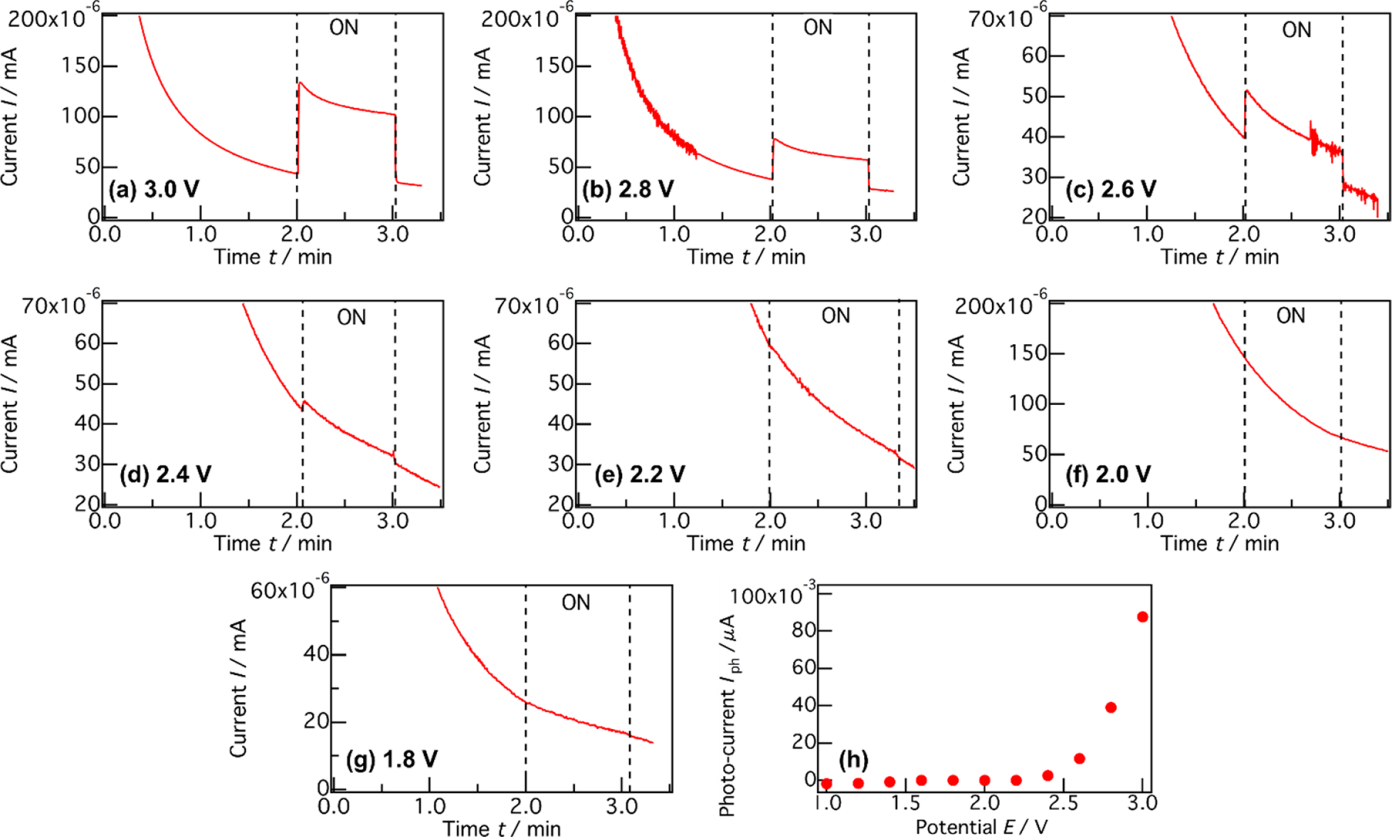
(a–g) CA curves at 1.8–3.0 V under dark/UV irradiation and (h) a photocurrent–voltage curve estimated from the CA curves of quartz/SRO/a-TiO_2_:Nb/LPO/Li. The CA curves at 1.0–1.6 V are in Figure S7.

In the case of photoelectrochemical cells using liquid electrolytes, photoelectrodes comprising n-type semiconductors are active at potentials that are more positive than those of the *E*_FB_ values under light irradiation because of the band bending toward negative potentials.^1−9^ Therefore, it is important to investigate the dependence of the photoelectrochemical reaction at the solid/solid interface on the applied voltage. The photocurrents at each voltage (Figures 3(a–g) and S7) are shown in Figure 3(h). Cathodic currents derived from discharging were confirmed under dark conditions in the range of 1.0–1.6 V because a-TiO_2_:Nb was located at a more negative potential than that of Li^+^ (de)intercalation (1.75 V).^35,36^ In contrast, anodic currents derived from charging were confirmed at >1.8 V. Continuous photocurrents were obviously confirmed at >2.6 V. The photoresponse was slightly confirmed even at 2.4 V. Therefore, the onset potential of the photocurrent was approximately 2.5 V, indicating that the onset potential of the photocurrent agreed approximately with the *E*_FB_ in Figure 1(c). This agreement suggests that the photoelectrochemical reaction at the solid/solid interface proceeded similarly to those at solid/liquid interfaces as below. Electrons in an electrode must migrate to a current collector for electrochemical oxidation. Below 2.4 V, the CBM bent toward a positive potential at the a-TiO_2_:Nb/LPO interface because a-TiO_2_:Nb was located at a more negative level than the *E*_FB_. Therefore, the photoexcited electrons cannot migrate to the current collector, leading to no photocurrent being generated. At >2.4 V, the CBM bent toward a negative potential with the increase in the applied bias because a-TiO_2_:Nb was located at more positive levels than its *E*_FB_. Bending toward a negative potential enabled the photoexcited electrons to migrate to the current collector, resulting in the generation of continuous photocurrents. Thus, we have obviously demonstrated that solid/solid interfaces consisting of semiconductor electrodes and solid ionic conductors can function in photoelectrochemical reactions similar to solid/liquid interfaces for the first time, as far as we know.

**Figure 4:**
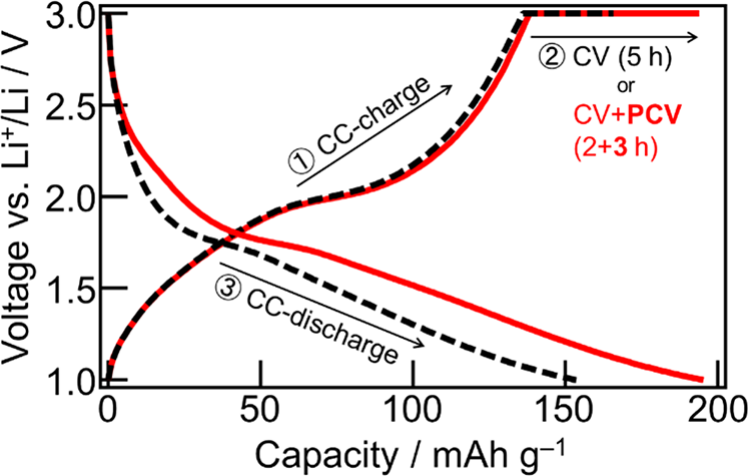
CC–CV–(PCV) charge/discharge curves of quartz/SRO/a-TiO_2_:Nb/LPO/Li. Both discharge measurements were performed under dark conditions after CC–CV–(PCV) charging.

The electrochemical charge–discharge reaction (Ti^4+^/Ti^3+^ + Li^+^-(de)intercalation) proceeded reversibly at the a-TiO_2_:Nb/LPO interface under the dark condition (Figure S5). The reversibility under light irradiation should also be investigated. Therefore, constant-current–constant-voltage (CC–CV) measurements of a-TiO_2_:Nb/LPO/Li were performed, as shown in Figure 4. The a-TiO_2_:Nb/LPO/Li cell was irradiated with light during additional constant–voltage (CV) charging for 3 h (PCV) after CV charging in the dark for 2 h. In the comparative dark test, the CV charge was measured for 5 h under the dark condition. Both discharges were performed under dark conditions after CC–CV–(PCV) charging. The charge capacity increased with light irradiation, suggesting that the photoelectrochemical reaction proceeded at the a-TiO_2_:Nb/LPO interface. The increase in the charge capacity might be because parts of the immobilized Li^+^ ions preintercalated during the LPO deposition were additionally deintercalated with the oxidation force of photogenerated holes. These results imply that Li^+^ diffusion in a-TiO_2_:Nb and/or Li^+^ transfer at the a-TiO_2_:Nb/LPO interface were promoted through accelerating the oxidation of Ti^3+^ to Ti^4+^ with the driving force of the photogenerated holes. The discharge capacity after the CC–CV–PCV charging was approximately the same as the CC–CV–PCV charge capacity. Moreover, a-TiO_2_:Nb/LPO/Li exhibited a high retention even under light irradiation (Figure S8). These results indicated that the photoelectrochemical oxidation proceeded at the a-TiO_2_:Nb/LPO interface with reversibility for electrochemical reduction in the dark. Therefore, the a-TiO_2_:Nb/LPO interface is considered to be stable for the photoelectrochemical reactions. The preintercalated Li^+^ might have partially remained in a-TiO_2_:Nb even after PCV charge. Therefore, Li^+^ deintercalated with photoenergy was able to be reversibly intercalated at CC discharge after PCV charge. These results might be because accelerating the oxidation of Ti^3+^ to Ti^4+^ by the light irradiation subsequently cause also improving Li^+^ diffusion within a-TiO_2_:Nb and/or Li^+^ transfer at the a-TiO_2_:Nb/LPO interface. In addition to these factors, we have to consider the difference in the potential between electrons and photogenerated holes, the effect of the quasi-Fermi level on the electrode potential, the change in the local electric field, diffusion barriers in the crystal structure, and so on. These factors might possibly contribute to Li^+^ transfer at an a-TiO_2_:Nb/LPO interface and/or Li^+^ diffusion within a-TiO_2_:Nb. However, we cannot judge which is the rate-determining step at the present stage. After the clarification of the detail principle, we will investigate which processes are improved by light irradiation.

**Figure 5:**
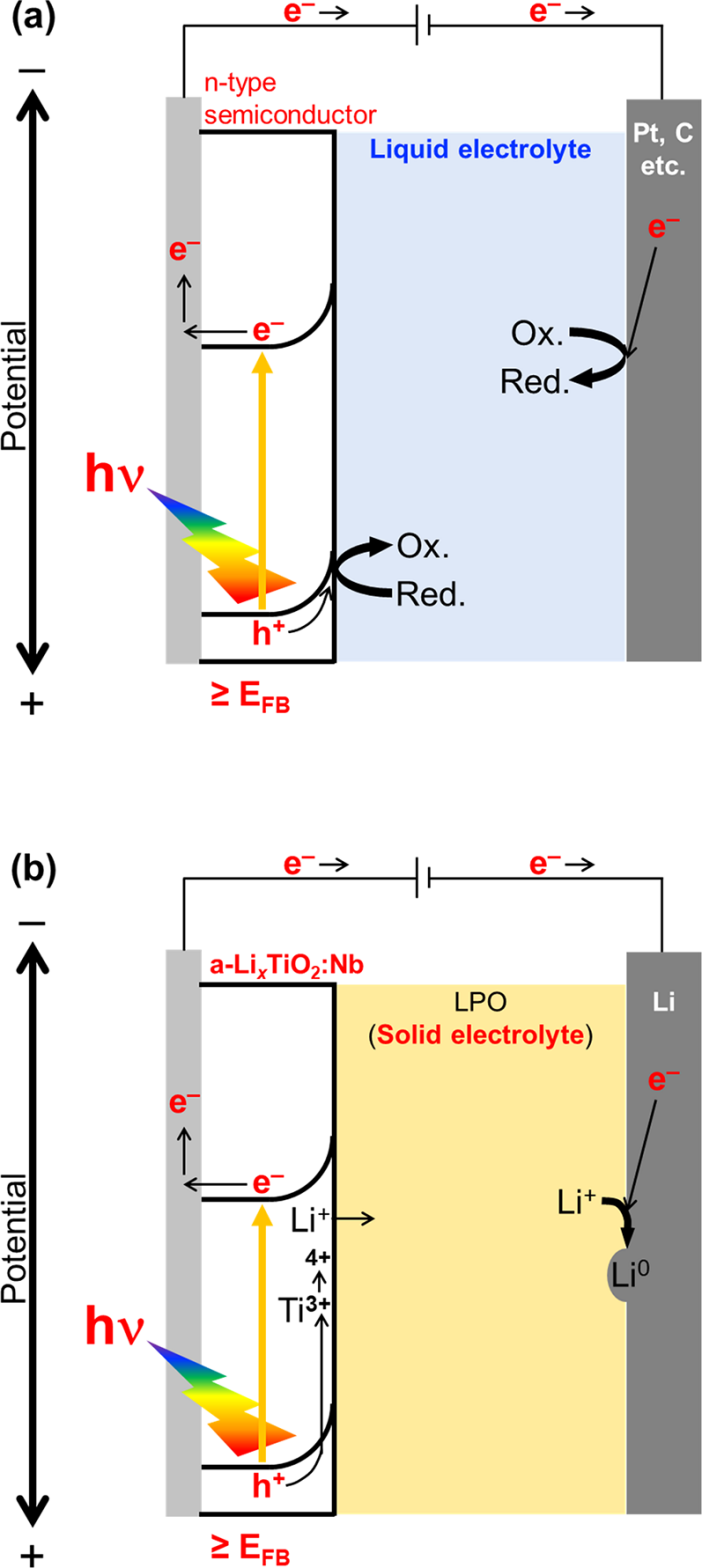
(a) The mechanism of the general photoelectrochemical cells using n-type semiconductor electrodes and liquid electrolytes. (b) The proposed mechanism of the photoelectrochemical reaction in a-TiO_2_:Nb/LPO/Li all-solid-state cell.

The proposed mechanism of the photoelectrochemical reaction in an a-TiO_2_:Nb/LPO/Li all-solid-state cell is described, and the mechanism of general photoelectrochemical cells using n-type semiconductor electrodes and liquid electrolytes is shown in Figure 5. Generally, electrochemical cells show photocurrents when the n-type semiconductor electrodes are located at potentials more positive than their *E*_FB_ because of their band bending toward negative potentials (Figure 5(a)). The photogenerated holes in the semiconductor electrodes are used for oxidation. The photoexcited electrons migrated to counter electrodes, such as Pt or C, and are used for reduction. In addition, a-TiO_2_:Nb/LPO/Li functioned similarly to general photoelectrochemical cells (Figure 5(b)). When the potential of a-TiO_2_:Nb was more positive than the *E*_FB_, the photoexcited electrons migrated to the current collector because of the CBM bending toward the negative potential, resulting in the generation of the continuous photocurrents. The photogenerated holes may oxidize Ti^3+^ into Ti^4+^, resulting in an increase in the charge capacity. The photoexcited electrons reduced Li^+^ to Li^0^ in the Li counter electrode. If photoexcited electrons are located at a more negative potential than the reaction potential of the counter electrode with keeping the band bending described in Figure 5(b), photoelectrochemical reactions would proceed without applied voltages. Therefore, photoelectrochemical reactions proceeded even at solid/solid interfaces comprising n-type semiconductor electrodes and solid electrolytes, similar to reactions at solid/liquid interfaces.^1−9^ Thus, we successfully demonstrated a stable photoelectrochemical reaction at the solid/solid interface.

There is no doubt about the possibility of stable photoelectrochemical reactions, even at solid/solid interfaces. Even if redox reactions do not proceed, the voltage dependence of photoresponse can be applied to photorechargeable supercapacitors. Moreover, there is also the possibility that this phenomenon contributes to dye-sensitized solar cells by changing liquid electrolytes to solid ones. Then, our results extend the research field of photoelectrochemistry to all-solid-state systems consisting of solid/solid interfaces. In addition, solid/solid interfaces can also be applied to analyses of phenomena that are hidden in information derived from decomposition of electrolytes and elution of electrodes because of unstable liquid electrolytes. Thus, we believe that this extension would enable us to investigate photoelectrochemical phenomena uncleared because of low stability and durability and that the photoelectrochemistry of all-solid-state systems will contribute to future solar energy conversion and storage.

In conclusion, we demonstrated a stable photoelectrochemical reaction at a semiconductor-electrode/solid-electrolyte interface in a-TiO_2_:Nb/LPO/Li all-solid-state cell. a-TiO_2_:Nb/LPO/Li was reversibly charged and discharged under dark conditions with high capacities. The oxidative currents of a-TiO_2_:Nb/LPO/Li increased upon light irradiation during the CA measurements when a-TiO_2_:Nb was located at a potential more positive than the *E*_FB_. This is because the photoexcited electrons were able to migrate to the current collector as the CBM bent toward the negative potential. The photoelectrochemical reaction at the semiconductor/solid-electrolyte interface was driven by the same principle as that at semiconductor/liquid-electrolyte interfaces. Moreover, oxidation under light irradiation exhibited reversibility with reduction under dark conditions. Thus, we extended the research field of photoelectrochemistry to all-solid-state systems composed of solid/solid interfaces. This extension would contribute to the development of photochemistry and solar energy utilization.

## Supporting Information Available

Fabrication conditions of the films, photographs of the photoelectrochemical measurement setup and a thin-film ASSB example, XRD patterns and Dektak profile of the obtained a-TiO_2_:Nb films, charge/discharge curves and capacity retention under dark conditions of ITO/a-TiO_2_:Nb/LPO/Li cell, temperature profile between the cell and the light source, CA curves of SRO/a-TiO_2_:Nb/LPO/Li at 1.0–1.6 V, and charge/discharge curves of SRO/a-TiO_2_:Nb/LPO/Li measured in CC-(P)CV modes (PDF)

## Abbreviations

a-TiO2: anatase TiO_2_
r-TiO2: rutile TiO_2_
LPO: amorphous Li_3_PO_4_
ITO: indium tin oxide
SRO: SrRuO_3_
CBM: conduction band minimum
VBM: valence band maximum
CC: constant current
CV: constant voltage
PCV: photoirradiated constant voltage
CA: chronoamperometry
