# Supplementary material for: Stable Photoelectrochemical Reactions at Solid/Solid Interfaces toward Solar Energy Conversion and Storage
Source: Nano Lett. 2024 Jan 12;24(6):1916–22. doi: 10.1021/acs.nanolett.3c03982 (PMC10870756; doi:10.1021/acs.nanolett.3c03982)
Supplement: Supplementary file 1 — nl3c03982_si_001.pdf [file nl3c03982_si_001.pdf]

Supporting information

Stable Photoelectrochemical Reactions at Solid/Solid Interfaces toward Solar Energy Conversion and Storage

Kenta Watanabe,*[1] Yuhei Horisawa,[1] Masataka Yoshimoto,[1] Kazuhisa Tamura,[3] Kota Suzuki,[2] Ryoji Kanno,[2] and Masaaki Hirayama*[1,2]

[1]Department of Chemical Science and Engineering, School of Materials and Chemical Technology, Tokyo Institute of Technology, 4259 Nagatsuta-cho, Midori-ku, Yokohama 226-8501, Japan

[2]Research Center for All-Solid-State Battery, Institute of Innovative Research, Tokyo Institute of Technology, 4259 Nagatsuta-cho, Midori-ku, Yokohama 226-8501, Japan

[3]Materials Sciences Research Center, Japan Atomic Energy Agency, 1-1-1 Koto, Sayo, Hyogo 679-5148, Japan

<u>**Contents**</u>

Experimental S2
Table S1~S3. Synthetic conditions of thin-film ASSBs S3
Figure S1. Overviews of an ASSB and the photoelectrochemical system. S4
Figure S2. XRR spectra of 30 nm-thick a-TiO_2:Nb film. S4
Figure S3. Synchrotron GI-XRD patterns of 30 nm-thick a-TiO_2:Nb(/ITO) film. S4
Figure S4. Dektak and XRD results of 400 nm-thick a-TiO_2:Nb film. S5
Figure S5. Charge/discharge profiles of a-TiO_2:Nb/LPO/Li in dark. S5
Figure S6. Temperature changes under light irradiation at various distances. S5
Figure S7. CA curves of a-TiO_2:Nb/LPO/Li at < 1.6 V under dark and light irradiation. S6
Figure S8. Charge/discharge profiles of a-TiO_2:Nb/LPO/Li under light irradiation. S6

EXPERIMENTAL

Sample Preparation

ITO/a-TiO_2:Nb/LPO/Li and SRO/a-TiO_2:Nb/LPO/Li thin-film all-solid-state cells were prepared on SiO_2 glass substrates by pulsed laser deposition, radio frequency magnetron sputtering, and vacuum vapor deposition under the conditions summarized in Tables S1–S3. ITO, a-TiO_2:Nb, LPO, and SRO are Indium Tin Oxide, Nb-doped a-TiO_2,[1–5] amorphous Li_3PO_4,[6–8] and $SrRuO_3$, respectively. ITO and SRO were used as current collectors. ITO was utilized for the Mott-Schottky analysis without any problem. However, SRO was applied to the photoelectrochemical measurements because ITO is possibly deactivated by heating. LPO was used as a solid electrolyte. The a-TiO_2 doped with 5% Nb, which possesses the enough electronic conductivity, was used as the target material for the deposition of a-TiO_2:Nb.

Characterization

The thickness of the obtained films was measured with stylus profiling (Bruker, DektakXT) and X-ray reflectometry (XRR, Rigaku; SmartLab). The crystal phases were identified with X-ray diffraction (XRD, Rigaku; ATX-G) using Cu $K\alpha_1$ of the incident beam from 10° to 60° every 0.02° at a rate of 5° min^{-1}. The grazing incidence X-ray diffraction (GI-XRD) patterns of a-TiO_2:Nb(/ITO)/SiO_2-glass were collected using a κ-type six-circle diffractometer (Newport Corporation, USA) installed at BL22XU of the synchrotron beam facilities SPring-8 in Japan. The X-rays were monochromated using a Si(111) double-crystal system. An X-ray wavelength of 0.82518 Å (15keV) was employed.

Electrochemical Measurements

Electrochemical measurements were performed using a potentiostat/galvanostat (Biologic; VSP-300). To estimate the E_{FB} of the a-TiO_2:Nb film, electrochemical impedance spectroscopy was conducted using ITO/a-TiO_2:Nb/LPO/Li at 10 mV of the amplitude with a sweeping frequency ranging from 10 mHz to 1 Mhz. The T_{CPE} values estimated from the Nyquist plots are usually converted to C_{scl} values using Equation (1) when a parallel *RCPE* is applied to equivalent circuits.

$$C_{scl} = T_{CPE}^{1/p} R^{(1-p)/p} \quad (1)$$

In Equation 1, C_{scl} is the capacitance of the space-charge layer in F, T_{CPE} is the CPE constant in F s^{p-1}, R is the resistance in Ω, and p is the CPE index.

Then, the estimated C_{scl} was used to create a Mott–Schottky plot, as shown in Equation (2):

$$C_{scl}^{-2} = 2(E - E_{FB} - kT/q)(e_0 e_S q N_D)^{-1} \quad (2)$$

where e_0 is the permittivity of a vacuum in F m^{-1}, e_S is the relative permittivity, q is the elementary charge ($1.6021773 \times 10^{-19}$ C), N_D is the carrier density in m^{-3}, E is the electrode potential in V, E_{FB} is the flat

band potential in V, k is the Boltzmann constant (1.380649 × 10^{-23} J K^{-1}), and T is the absolute temperature in K. In the present study, *RCPE* parallel circuits could not be applied to all the Nyquist plots. Therefore, a Mott–Schottky plot was obtained using T_{CPE} values instead of C_{scl} values, which was used to estimate the E_{FB}.[9,10] The difference between the T_{CPE} and C_{scl} affects the slopes of the fitting lines, whereas the intercepts remain relatively unchanged.[9,10] Slopes of Mott–Schottky plots are used to estimate the career densities. E_{FB} is estimated from an intercept of Mott–Schottky plots. Thus, the E_{FB} of the obtained a-TiO_2:Nb film was estimated from the intercept on the x-axis (potential/V) of the Mott–Schottky plot using T_{CPE}.

The charge/discharge properties were measured in the CC-CV mode. The photo-response was investigated using light irradiation during CV charging (PCV) after CC or CC-CV charging in the dark using the system shown in Figure S1. An LED (OptoCode; LED365 – SPT/L, NICHIA; NCSU033B) with peak tops at 365 and 10 nm of the FWHM was used as a light source. The photoresponse was also investigated using the CA mode (CV charge) at various voltages. The CA measurements started after the cell voltages reached the measurement voltages during CC charging. The change in the current upon turning on the LED was measured during the CA tests under dark conditions.

Table S1. Synthesis conditions for Nb-doped anatase-TiO_2 (a-TiO_2:Nb) films on ITO or SRO/quartz substrates using pulsed laser deposition (PLD)

Laser energy		Lens / mm	Target–substrate distance / mm	Temperature / K	Gas		Laser frequency / Hz
/ eV	/ KV				Atmosphere	Pressure / Pa	
150	24	–20	84	773	O_2	15	5

ITO: Indium Tin Oxide, SRO: $SrRuO_3$

Table S2. Synthesis conditions for SRO films on quartz glass substrates using PLD

Laser energy		Lens / mm	Target–substrate distance / mm	Temperature / K	Gas		Laser frequency / Hz
/ eV	/ KV				Atmosphere	Pressure / Pa	
150	24	–40	84	773	O_2	15→15→50	5

Table S3. Synthesis conditions for amorphous Li_3PO_4 (LPO) on a-TiO_2:Nb films by radio frequency (RF) magnetron sputtering

Target	Energy / W	Time / h	Temperature	Target–substrate distance / mm	Gas	Gas flow / sccm	O_2 pressure / Pa
Li_3PO_4	100	5	R.T.	30	Ar / O_2 (1 / 4)	5 / 20	8.0→0.51

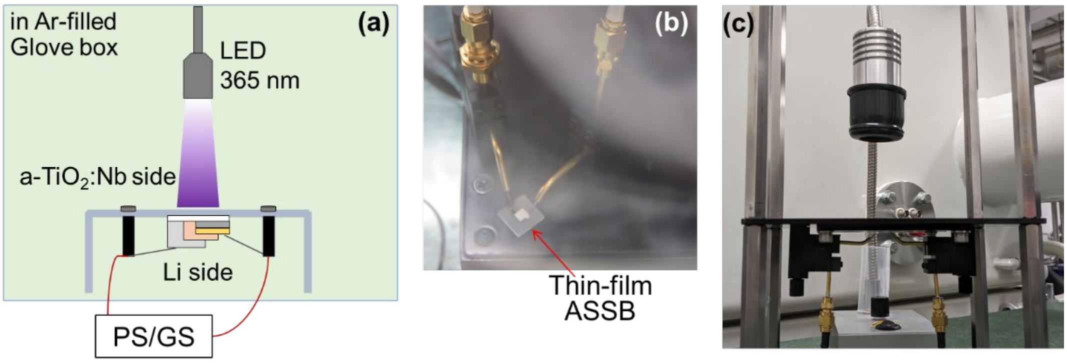

Figure S1. Overview of the thin-film ASSB and the system for photoelectrochemical measurements. The stage of (c) is updated from that of the setup used in the manuscript.

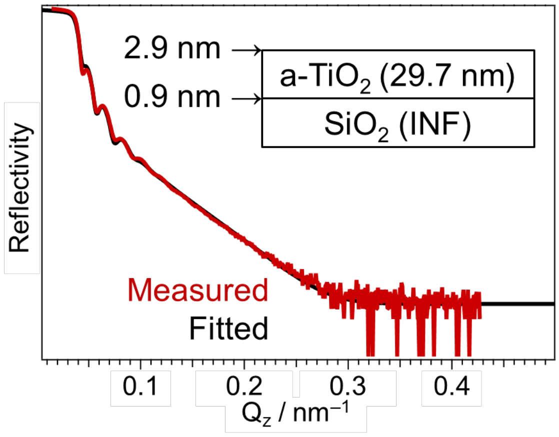

Figure S2. Measured and fitted XRR spectra of a-TiO_2:Nb/SiO_2-glass deposited at 773 K.

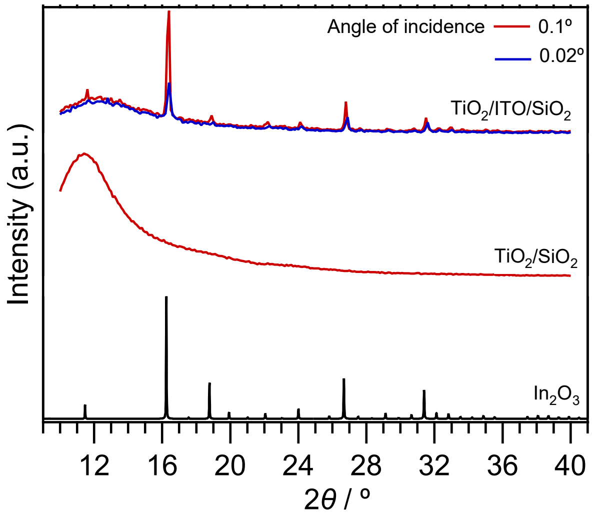

Figure S3. Synchrotron GI-XRD patterns of a-TiO_2:Nb/SiO_2-glass and a-TiO_2:Nb/ITO/SiO_2-glass measured at the beam line BL22XU in Spring-8. a-TiO_2:Nb were deposited at 773 K.

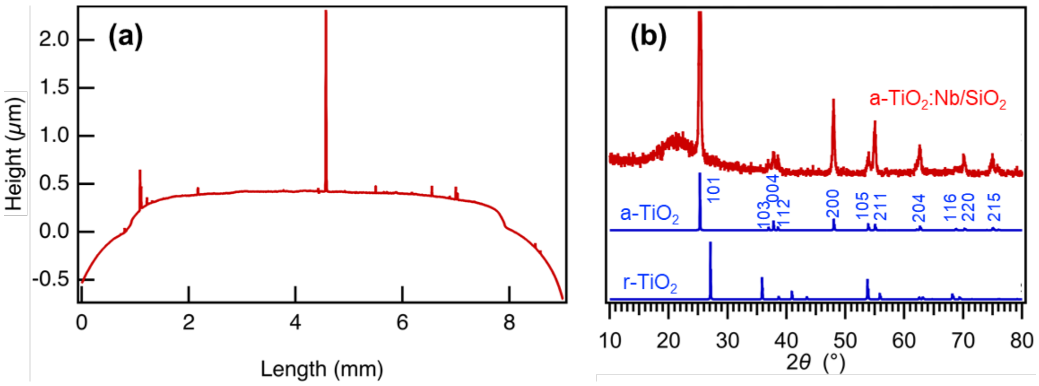

Figure S4. (a) The Dektak profile and (b) XRD pattern of a thick a-TiO_2:Nb film deposited on a SiO_2-glass substrate at 773 K.

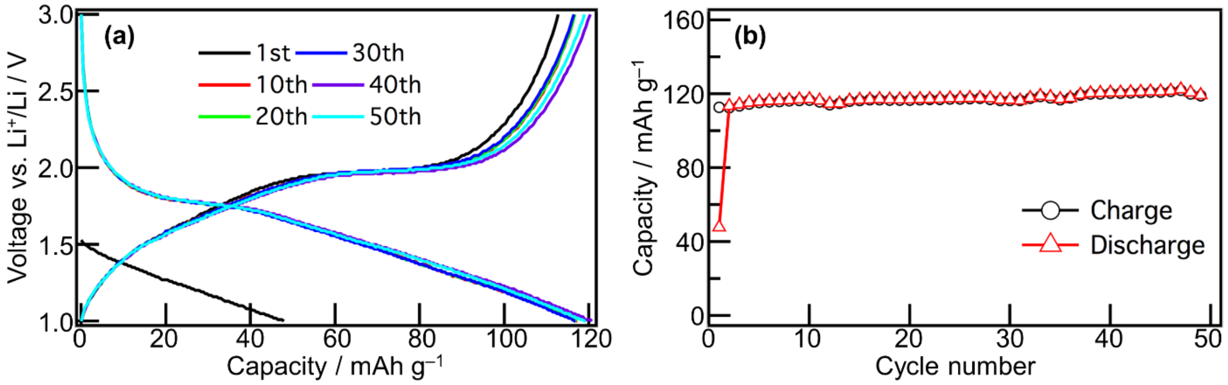

Figure S5. (a) Charge/discharge curves and (b) the capacity retention of quartz/ITO/a-TiO_2:Nb/LPO/Li under dark conditions. The a-TiO_2:Nb films were prepared at 773 K.

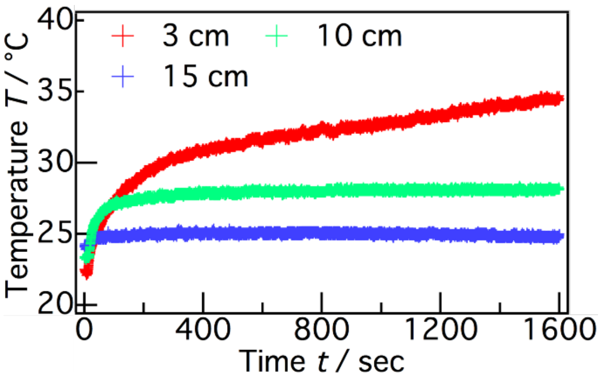

Figure S6. Temperature changes under light irradiation at distances of 3, 10, and 15 cm.

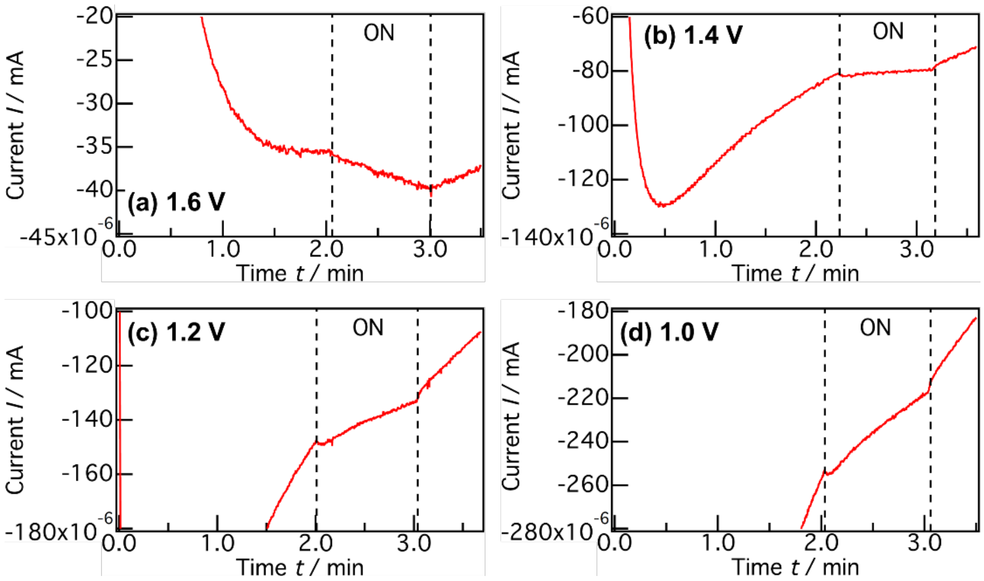

Figure S7. CA curves of quartz/SRO/a-TiO_2:Nb/LPO/Li at 1.0–1.6 V under dark and light irradiation.

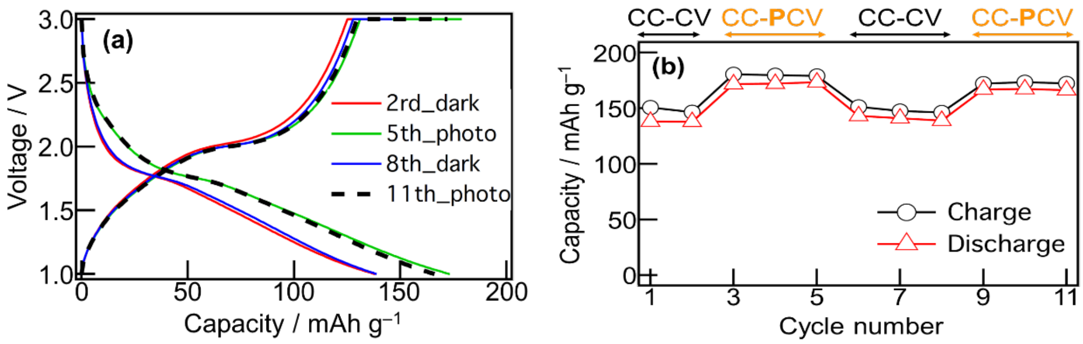

Figure S8. Charge/discharge curves of quartz/SRO/a-TiO_2:Nb/LPO/Li measured in CC-(P)CV modes. One, two, and six to eight cycles were performed in the dark. Three to five and nine to eleven cycles were conducted with CC-(P)CV charge. (P)CV charge was performed for 3 h after CC charge. All discharges were measured in the dark.

References

(1) Mulmi, D. D.; Sekiya, T.; Kamiya, N.; Kurita, S.; Murakami, Y.; Kodaira, T. Optical and Electric Properties of Nb-Doped Anatase TiO_2 Single Crystal. *J. Phys. Chem. Solids* **2004**, *65* (6), 1181–1185.

(2) Furubayashi, Y.; Hitosugi, T.; Yamamoto, Y.; Inaba, K.; Kinoda, G.; Hirose, Y.; Shimada, T.; Hasegawa, T. A Transparent Metal: Nb-Doped Anatase TiO_2. *Appl. Phys. Lett.* **2005**, *86* (25), 252101.

(3) Furubayashi, Y.; Hitosugi, T.; Yamamoto, Y.; Hirose, Y.; Kinoda, G.; Inaba, K.; Shimada, T.; Hasegawa, T. Novel Transparent Conducting Oxide: Anatase $Ti_{1-x}Nb_xO_2$. *Thin Solid Films* **2006**, *496* (1), 157–159.

(4) Hitosugi, T.; Ueda, A.; Nakao, S.; Yamada, N.; Furubayashi, Y.; Hirose, Y.; Shimada, T.; Hasegawa, T. Fabrication of Highly Conductive $Ti_{1-x}Nb_xO_2$ Polycrystalline Films on Glass Substrates via Crystallization of Amorphous Phase Grown by Pulsed Laser Deposition. *Appl. Phys. Lett.* **2007**, *90* (21), 212106.

(5) Hitosugi, T.; Ueda, A.; Furubayashi, Y.; Hirose, Y.; Konuma, S.; Shimada, T.; Hasegawa, T. Fabrication of TiO_2-Based Transparent Conducting Oxide Films on Glass by Pulsed Laser Deposition. *Jpn. J. Appl. Phys.* **2007**, *46* (1L), L86.

(6) Bates, J. B.; Dudney, N. J.; Gruzalski, G. R.; Zuhr, R. A.; Choudhury, A.; Luck, C. F.; Robertson, J. D. Fabrication and Characterization of Amorphous Lithium Electrolyte Thin Films and Rechargeable Thin-Film Batteries. *J. Power Sources* **1993**, *43* (1), 103–110.

(7) Kuwata, N.; Iwagami, N.; Tanji, Y.; Matsuda, Y.; Kawamura, J. Characterization of Thin-Film Lithium Batteries with Stable Thin-Film Li_3PO_4 Solid Electrolytes Fabricated by ArF Excimer Laser Deposition. *J. Electrochem. Soc.* **2010**, *157* (4), A521.

(8) Kuwata, N.; Kudo, S.; Matsuda, Y.; Kawamura, J. Fabrication of Thin-Film Lithium Batteries with 5-V-Class $LiCoMnO_4$ Cathodes. *Solid State Ionics* **2014**, *262*, 165–169.

(9) Harrington, S. P.; Devine, T. M. Analysis of Electrodes Displaying Frequency Dispersion in Mott-Schottky Tests. *J. Electrochem. Soc.* **2008**, *155* (8), C381.

(10) Kurtz, M. A.; Wessinger, A. C.; Taylor, L. M.; Gilbert, J. L. Electrode Potential, Inflammatory Solution Chemistry and Temperature Alter Ti-6Al-4V Oxide Film Properties. *Electrochim. Acta* **2023**, *462*, 142770.
